# Dietary Fiber Improves Somatosensory Function in Western Diet–Fed Mice by Remodeling Adipose Immune Cells via FFAR2 Signaling

**DOI:** 10.21203/rs.3.rs-7868021/v1

**Published:** 2025-11-03

**Authors:** Chaitanya Gavini, Laetitia Raux, Gwenaël Labouèbe, Emily Gornick, Sarah Mc Hugh, Nadia Elshareif, Nigel Calcutt, Pietro Di Summa, François Gorostidi, Pascale Vonaesch, Virginie Mansuy-Aubert

**Affiliations:** University of Lausanne; University of Lausanne; University of Lausanne; Loyola University Chicago; University of Lausanne; University of Lausanne; University of California, San Diego; University Hospital of Lausanne; University Hospital of Lausanne; University of Lausanne; University of Lausanne

**Keywords:** Dietary fiber, Microbiome, Metabolism, Neuroinflammation, Gut microbiome, Inulin, Microbiome-Gut-adipose-PNS axis, Peripheral nerve function

## Abstract

Westernized diets (WDs)—high in fat and sugar and low in fiber—produce somatosensory deficits, chronic pain, and neuropathy, yet the mechanisms linking diet to peripheral nervous system (PNS) pathology remain incompletely defined. Emerging evidence implicates gut-derived metabolites in sensory homeostasis; for example, fecal microbiota transplantation (FMT) from lean donors to WD fed mice reduces hypersensitivity and attenuates PNS inflammation, although FMT outcomes are variable. We therefore tested whether targeted modulation of the gut microbiota with fermentable complex carbohydrates could reproducibly improve somatosensory function in WD-fed mice. Using an integrated pipeline—behavioral and physiological assays, peripheral nerve electrophysiology, and molecular and immune profiling—we show that short-chain fatty acids (SCFAs) generated by fermentation remodel adipose tissue depots and act via the SCFA receptor FFAR2 (GPR43) to ameliorate sensory deficits. These findings identify a microbiota–SCFA–FFAR2 axis that couples dietary fiber to PNS function and provide a tractable alternative to FMT for mitigating WD-associated sensory neuropathy.

## Introduction

Obesity and Western-style diets (WDs), characterized by excess fat and sugar and reduced fiber intake, are major global health concerns that increase the risk of metabolic disease, cardiovascular disease, and cancer^1,2^. Less recognized is their impact on the peripheral nervous system (PNS): individuals with obesity or type 2 diabetes frequently develop chronic pain, sensory loss, and neuropathy^3–6^.

Somatosensory dysfunction is an underappreciated complication of obesity and poor dietary habits. Traditionally associated with diabetic neuropathy, sensory deficits are increasingly recognized in individuals exposed to high-calorie, low-fiber diets even in the absence of hyperglycemia^7–9^. These observations suggest that diet-induced alterations in systemic and tissue homeostasis contribute to sensory pathology through mechanisms distinct from classical diabetic neuropathy^10–12^.

Emerging evidence points to the gut microbiota as a key mediator of metabolic and neurological outcomes. Fecal microbiota transplantation (FMT) from lean donors to WD fed mice reduces hypersensitivity and attenuates PNS inflammation, although FMT outcomes are variable^13–17^. We therefore tested whether targeted modulation of the gut microbiota with fermentable complex carbohydrates could reproducibly improve somatosensory function in WD-fed mice. Fermentable fibers such as inulin are metabolized by intestinal bacteria to produce short-chain fatty acids (SCFAs), including acetate, propionate, and butyrate^18–20^. SCFAs regulate host metabolism, and immune function, and have been implicated in the gut–brain axis^18–21^. However, whether microbial metabolites directly influence peripheral somatosensory neurons or act indirectly through immune or metabolic pathways remains unresolved.

Here, we investigated whether dietary inulin supplementation could prevent or reverse WD-induced somatosensory dysfunction. Using behavioral, electrophysiological, immunological, and transcriptomic approaches, we show that inulin attenuates sensory hypersensitivity not through direct neuronal effects of SCFAs, but by remodeling adipose tissue immunity and reducing systemic inflammation. Using cell-specific approaches, we identify myeloid FFAR2 as a critical mediator of this process, linking gut microbiota-derived metabolites to adipose and sensory tissue immune reprogramming. These findings establish a gut–adipose–nerve axis as a central pathway by which dietary fiber preserves sensory function in the context of an unhealthy diet.

## Results

### Inulin attenuates WD-induced somatosensory hypersensitivity and neuronal hyperexcitability

**Figure 1:**
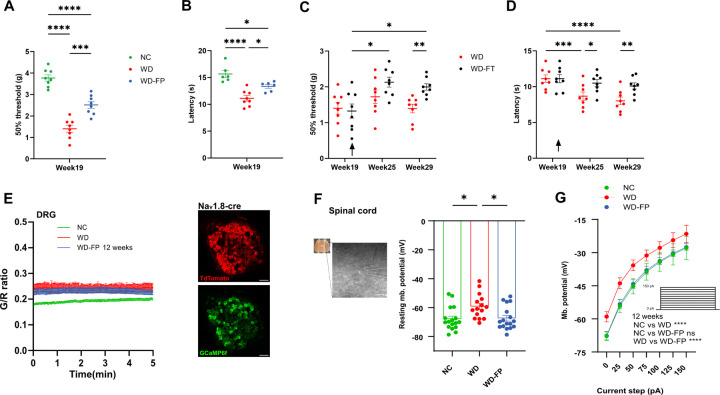
Inulin supplementation mitigates Western diet–induced somatosensory hypersensitivity and reduces neuronal hyperexcitability. **A–B)** Mechanical sensitivity (**A**) and thermal sensitivity (**B**) assessed by the von Frey and Hargreaves tests respectively at 19 weeks (after 12 weeks of diet) in mice fed normal chow (NC), Western diet (WD), or WD supplemented with 10% inulin from the beginning (WD-FP, prevention group); *n*=8/group. **C–D)** Mechanical (**C**) and thermal sensitivity (**D**) in mice fed Western diet (WD), or WD supplemented with 10% inulin after 12 weeks of WD (WD-FT, treatment group); *n*=8/group. Arrow represents timepoint where mice on WD diet shift to WD supplemented with 10% inulin. **E)** DRG neuronal activity assessed by calcium imaging: baseline G/R fluorescence ratio; *n*=9–10 ganglia/group. **F-G)** Resting membrane potential (**F**), and neuronal excitability assessment (current-clamp protocol, see insert) (**G**) in whole-cell patch-clamp recordings of substantia gelatinosa neurons in spinal cord slices following 14–18 weeks on diet; *n*=15–17 neurons/group. All data are presented as mean ± S.E.M. *p<0.05, **p<0.005, ***p<0.0005, ****p<0.00005. NC = normal chow; WD = Western diet; WD-FP = WD + 10% inulin (prevention); WD-FT = WD for 12 weeks followed by inulin (treatment).

Mice fed a Western diet (WD) for 12 weeks developed robust somatosensory dysfunction, with reduced paw withdrawal thresholds in the von Frey test and shortened latencies in the Hargreaves test compared to normal chow (NC) controls (Fig. 1A, B). Preventive inulin supplementation (WD-FP) partially rescued both mechanical and thermal sensitivity at this early stage. In a separate cohort, therapeutic inulin intervention initiated after 12 weeks of WD (WD-FT) progressively improved sensory thresholds, indicating that established deficits remained reversible (Fig. 1C, D). Electrophysiological recordings from dorsal root ganglia (DRG) neurons and in spinal cord revealed depolarized resting potentials and exaggerated excitability in WD-fed mice, both of which were partially normalized by inulin (Fig. 1E–G, Supp. 2A–C). These findings establish that dietary inulin both prevents and reverses WD-induced somatosensory dysfunction.

### Inulin restores systemic metabolic and microbial alterations induced by WD

**Figure 2:**
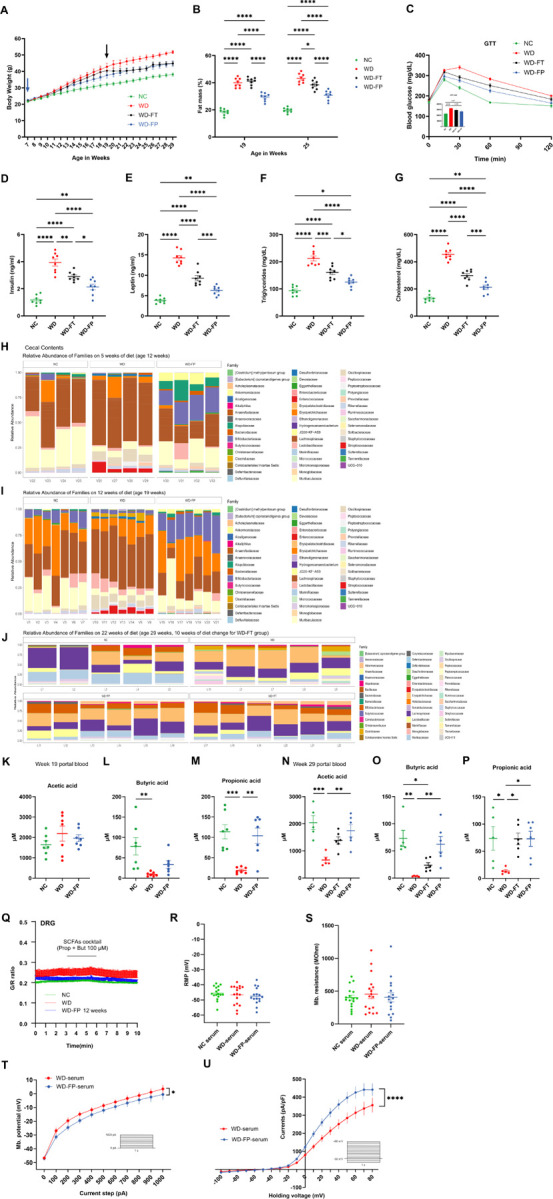
Inulin restores systemic metabolic and microbial alterations induced by WD, while serum factors modulate DRG neuronal excitability. **A–B)** Body weight (**A**) and fat mass percentage (**B**) of mice fed NC, WD, WD-FP, or WD-FT over 22 weeks; *n*=8/group. Arrow represents timepoint where mice on WD diet shift to WD supplemented with 10% inulin. **C)** Glucose tolerance test of NC, WD, WD-FP, or WD-FT mice; *n*=8/group. **D-G)** Circulating levels of insulin (**D**), leptin (**E**), triglycerides (**F**), and cholesterol (**G**); *n*=8/group. **H-J)** Relative abundance of bacterial families in the cecal contents assessed by 16S rRNA gene amplicon sequencing at weeks 5 (**H**), 12 (**I**), and 22 (**J**) of dietary intervention; *n*=4–6/group. **K–P**) SCFA levels (acetic, butyric, and propionic acid) in portal vein serum at 19 weeks (**H**) and 29 weeks (**I**); *n*=5–7/group. **Q)** DRG neuronal activity assessed by calcium imaging, G/R fluorescence ratio after SCFA application; *n*=9–10 ganglia/group. **R–S)** Resting membrane potential (**R**) and membrane resistance measurements (**S**) in DRG neurons exposed to serum from NC, WD, or WD-FP groups; *n*=16–17 cells/group **T)** Neuronal excitability assessment (current-clamp protocol) in neurons exposed to WD or WD-FP serum. Inset: current steps protocol; *n*=16–17 cells/group **U)** Current–voltage (I–V) relationship measured in DRG neurons incubated with WD vs WD-FP serum; Inset: voltage steps protocol; *n*=16–17 cells/group All values are mean ± S.E.M; significance indicators as above.

WD feeding caused accelerated weight gain, increased fat mass, glucose intolerance, and dyslipidemia compared to NC-fed controls (Fig. 2A–G). Inulin supplementation reduced adiposity and improved glucose tolerance in both preventive and therapeutic paradigms. Microbiome profiling revealed reduced diversity and depletion of fiber-degrading taxa (Lachnospirales, Bacteroidales) in WD-fed mice, with partial restoration by inulin (Fig. 2H–J, Supp. 1D–I). Consistent with these shifts, WD reduced cecal and portal vein concentrations of butyrate and propionate, while inulin supplementation restored their levels, with stronger effects at later timepoints (Fig. 2K–P, Supp. 1J–O). Thus, inulin counteracts WD-induced metabolic deterioration and restores microbial SCFA production.

### Serum factors, but not SCFAs, modulate DRG neuronal excitability in a diet-dependent manner

**Figure 3:**
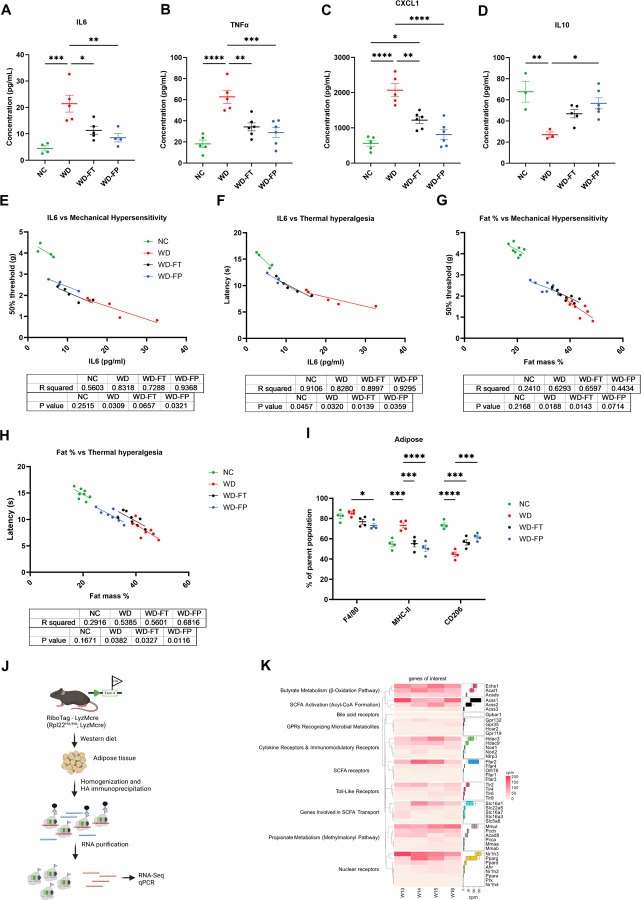
Inulin modulates adipose–immune–sensory axis via cytokine remodelling. **A–D)** Circulating pro-inflammatory (e.g., IL-6, TNFα, CXCL1) and anti-inflammatory (IL-10) cytokine profiles; *n*=4–6/group. **E–F)** Correlation analyses between IL6 and sensory outcomes: IL6 vs. mechanical and thermal thresholds. **G-H)** Correlation analyses between fat mass % and sensory outcomes: fat % vs. mechanical and thermal thresholds. **I)** Flow cytometry quantification of F4/80^+^ macrophage subtypes (MHC-II^+^pro-inflammatory and CD206^+^anti-inflammatory) in adipose after 22 weeks on diet (29 weeks of age); *n*=4/group. **J)** Schematic of RiboTag procedure. **K)** Translatomic snapshot of active immune pathways in adipose tissue myeloid cells after WD; *n*=4. All values are mean ± S.E.M; significance indicators as above.

To test whether SCFAs directly regulate peripheral sensory neurons, cultured DRG nociceptors were stimulated with SCFAs. Neuronal excitability was unchanged, indicating SCFAs alone do not directly modulate neuronal firing (Fig. 2Q). In contrast, DRG neurons exposed to serum from WD-fed mice displayed heightened excitability, while WDI serum enhanced outward (likely K^+^) currents (Fig. 2R–U). Cytokine profiling revealed a WD-induced pro-inflammatory signature (↑ IL-6, TNFα, CXCL1; ↓ IL-10), which was shifted toward an anti-inflammatory profile by inulin, especially under the preventive paradigm (Fig. 3A–D, Supp. 1P–U). Importantly, paw withdrawal thresholds correlated inversely with cytokines levels and adiposity (Fig. 3E–H), suggesting that circulating inflammatory mediatorslink diet to altered sensory function.

### Inulin remodels adipose tissue immunity and systemic cytokines to modulate sensory function

To identify the source of systemic inflammatory signals, we profiled immune cells in adipose tissue. WD feeding induced a pro-inflammatory immune landscape, whereas inulin supplementation promoted an anti-inflammatory remodeling of CD45^+^ adipose immune populations (Fig. 3I). These changes paralleled improvements in sensory thresholds, suggesting that adipose-derived cytokines influence neuronal function. Together, these results indicate that inulin protects against WD-induced somatosensory dysfunction not by acting directly on neurons, but by reprogramming adipose tissue immunity and systemic inflammatory tone.

### Adipose myeloid FFAR2 mediates gut–immune crosstalk underlying sensory dysfunction

**Figure 4:**
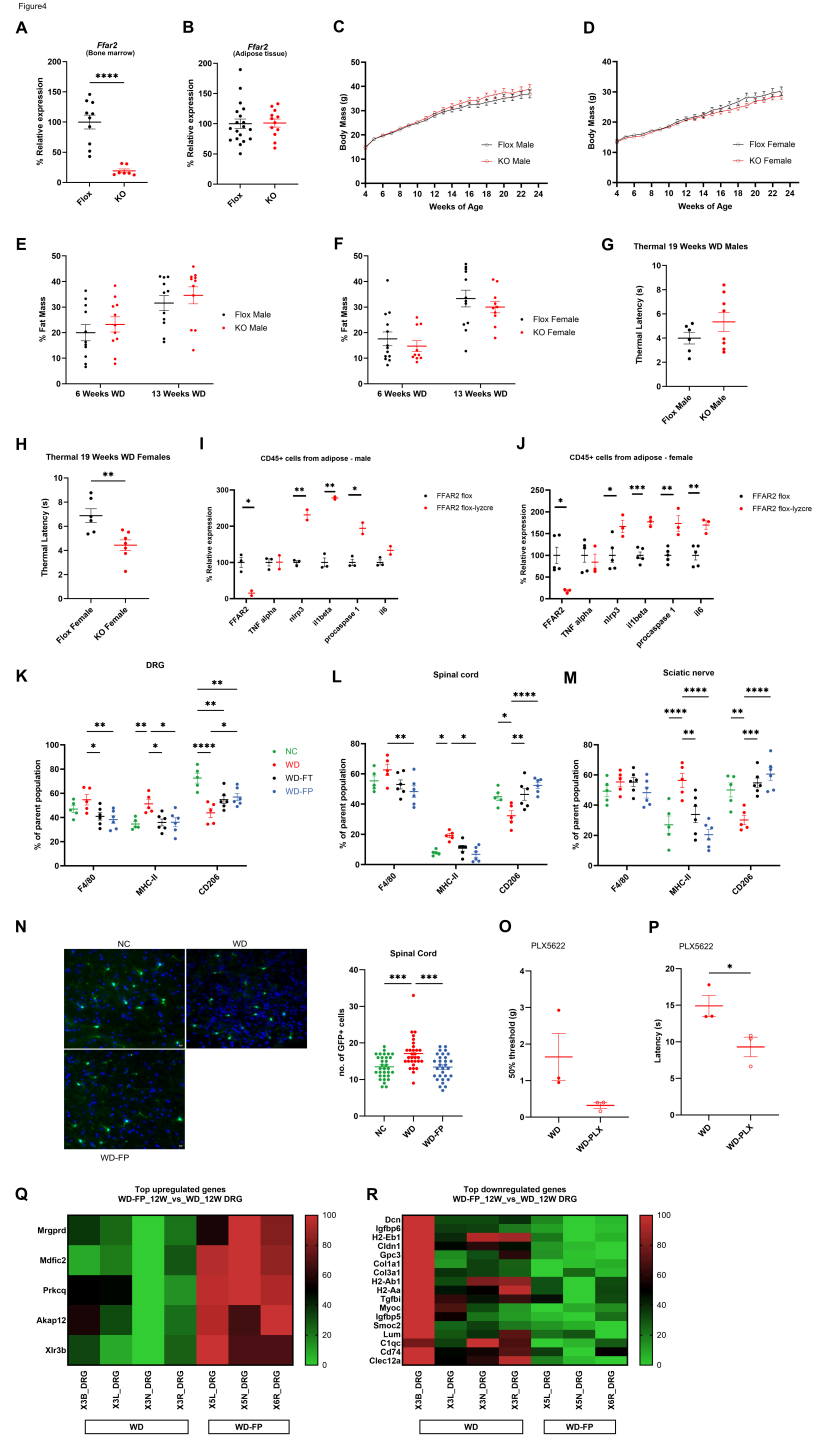
FFAR2 expression in myeloid cells regulates inflammation and sensory dysfunction. **A–B)**Tissue-specific deletion in FFAR2-LysMcre mice in bone marrow (**A**) and adipose (**B**); *n*=7– 19/group. **C–D)** Body weights of male (**C**) and female (**D**) FFAR2-Flox and FFAR2-KO mice fed WD; *n*=10–13/group. **E–F)** Fat mass of male (**E**) and female (**F**) FFAR2-Flox and FFAR2-KO mice fed WD; *n*=10–13/group. **G–H)** Thermal sensation in FFAR2-KO mice fed WD; *n*=5–8/group. **I–J)** Inflammatory gene expression (e.g., *Il1b, Il6, Nlrp3*) in CD45^+^ adipose immune cells from FFAR2-KO mice; *n*=2–5/group. **K–M)** Flow cytometry quantification of F4/80^+^ macrophage subtypes (MHC-II^+^pro-inflammatory and CD206^+^anti-inflammatory) in DRG (**K**), spinal cord (**L**), and sciatic nerve (**M**) after 22 weeks on diet (29 weeks of age); *n*=5–6/group. **N)** Representative images and quantification of CX3CR1-GFP^+^ immune cells infiltrating the spinal cord in NC vs. WD vs. WD-FP mice after 12 weeks on diet (19 weeks of age); *n*=3 mice/group. scale bar = 10 μm **O–P)** Behavioural hypersensitivity in WD-fed ± PLX5622 treatment; *n*=3/group. All data are presented as mean ± S.E.M. significance indicators as above. WD-PLX = WD mice treated with PLX5622. **Q–R)** Heatmaps of significantly upregulated (**Q**) and downregulated (**R**) genes in DRG’s of WD-FP vs. WD mice; *n*=3–4/group. All data are mean ± S.E.M; statistical significance as above. Flox = FFAR2-flox; KO = FFAR2-LysMcre.

To determine how gut-derived metabolites shape adipose immunity, we analyzed the translatome of adipose myeloid cells using RiboTag-LysMcre mice (Fig. 3J). WD-fed animals displayed transcriptional upregulation of SCFA metabolism (Ech1, Acaa1b, Acads), SCFA activation (Acss1–3), and microbial metabolite receptors (Ffar2, Gpr35, Gpr132), along with pro-inflammatory genes (Nod1, Nod2, Nlrp3) (Fig. 3K). Myeloid-specific deletion of Ffar2 confirmed its functional role: KO mice exhibited exaggerated inflammatory gene expression (Il1b, Nlrp3, Il6, Casp1) in adipose tissue and worsened heat hypersensitivity, particularly in females (Fig. 4A–J). Human adipose single-cell datasets confirmed FFAR2 expression in myeloid cells, while DRG and nerve immune populations lacked SCFA receptor expression (Supp. 2). These findings demonstrate that adipose myeloid FFAR2, but not neuronal FFAR2, mediates gut–immune–nerve communication.

### Inulin modulates neuroimmune interactions in sensory tissues to improve neuronal function

We next examined whether neuroimmune interactions in sensory tissues contribute to inulin’s protective effects. Flow cytometry revealed that WD feeding increased pro-inflammatory macrophages (MHC-II^+^) and reduced anti-inflammatory macrophages (CD206^+^) in DRG, spinal cord, and sciatic nerve (SN), while inulin supplementation restored a more anti-inflammatory profile (Fig. 4K–M). CX3CR1^+^ macrophage-like cells were also increased in WD spinal cord and reduced by inulin (Fig. 4N). Pharmacological depletion of macrophages with PLX5622 worsened hypersensitivity in WD-fed mice (Fig. 4O, P), confirming their modulatory role. Consistently, DRG RNA-seq revealed that inulin upregulated neuronal homeostasis genes (Mrgprd, Akap12) and downregulated pro-inflammatory/ECM genes (Dcn, H2-Eb1) (Fig. 4Q, R). Thus, inulin promotes both systemic (adipose) and local (DRG/spinal cord) immune remodeling to support sensory neuron function.

## Discussion

Somatosensory dysfunction is an underrecognized complication of unhealthy diets, occurring independently of hyperglycemia^3,4^. Here we identify a gut–adipose–nerve axis through which dietary inulin supplementation attenuates WD-induced sensory deficits. Rather than acting directly on sensory neurons, microbiota-derived metabolites regulate sensory function indirectly by remodeling adipose tissue immunity and systemic inflammation. We pinpoint myeloid FFAR2 as a critical mediator of this process, positioning adipose tissue as an active immune organ that relays microbial signals to the nervous system.

Our work clarifies an important mechanistic distinction: SCFAs restored by inulin feeding do not alter DRG neuronal excitability directly, consistent with the absence of SCFA receptor expression in sensory neurons. Instead, serum from WD-fed mice increased DRG excitability, coinciding with elevated circulating pro-inflammatory cytokines such as IL-6 and TNFα. This suggests that systemic factors derived in part from inflamed adipose depots mediate the neuronal effects of diet. Inulin supplementation normalized the cytokine milieu and restored sensory thresholds, underscoring the importance of systemic immune balance in diet-induced somatosensory dysfunction.

Adipose tissue emerged as a central hub linking gut microbial metabolism to neuronal health^22,23^. WD feeding drove pro-inflammatory remodeling of adipose immune cells, while inulin shifted these populations toward anti-inflammatory states. Translatomic profiling of adipose myeloid cells revealed SCFA metabolism and sensing pathways, including FFAR2, together with pro-inflammatory signatures under WD. Myeloid-specific FFAR2 deletion amplified inflammation and worsened sensory hypersensitivity, particularly in females, highlighting a sex-dimorphic contribution of adipose immunity to neuronal outcomes. These results converge with human adipose single-cell datasets, which confirmed enrichment of FFAR2 in myeloid cells but not in neurons or peripheral nerve immune cells, reinforcing the primacy of adipose immunity in gut–nerve communication.

Local neuroimmune interactions within sensory tissues also contributed to the phenotype^24,25^. WD feeding induced pro-inflammatory macrophage phenotypes in DRG, spinal cord, and sciatic nerve, while inulin supplementation restored a more homeostatic balance. Pharmacological depletion of macrophages exacerbated sensory hypersensitivity, demonstrating that resident immune cells modulate neuronal function^26,27^. RNA sequencing further revealed that inulin treatment upregulated genes involved in neuronal homeostasis and downregulated extracellular matrix and pro-inflammatory programs. Together, these findings suggest that adipose-derived signals set the systemic inflammatory tone, which is then integrated by local immune–neuron interactions in sensory tissues to shape excitability and function.

This study expands the concept of the gut–brain axis to include a gut–adipose–nerve axis, where dietary fiber intake restores microbial metabolite production, reprograms adipose myeloid immunity via FFAR2, and secondarily modulates local neuroimmune interactions in sensory tissues^28,29^. The discovery that FFAR2 signaling in adipose myeloid cells, rather than in neurons, is central to sensory protection emphasizes the importance of peripheral immune hubs in linking diet and neuronal health.

From a translational perspective, these findings suggest that dietary fiber supplementation, or strategies that mimic its effects on microbiota and myeloid FFAR2 activation, may provide novel approaches to mitigate somatosensory dysfunction associated with obesity and Western diet consumption. The observation of sex-specific effects of FFAR2 deletion also raises important questions about immune–metabolic dimorphism that warrant future investigation.

## EXPERIMENTAL MODEL AND SUBJECT DETAILS

### Mice Strains

Animal studies were conducted in accordance with the recommendations of the Guide for the Care and Use of Laboratory Animals of the National Institutes of Health and with the approval of the Loyola University Chicago Institutional Animal Care and Use Committee and by the ‘Service de la consommation et des Affaires vétérinaires’ (SCAV) of the Canton de Vaud in Switzerland. C57BL/6J (#000664), RiboTag (#011029), and LysMcre (#004781) were obtained from Jackson laboratory (Maine, USA). CX_3_CR1^EGFP^ mice line was kindly shared by the laboratory of Dr. Paolicelli, University of Lausanne. Ffar2 flox mice on a C57BL/6J background were generously provided by Dr. Brian Layden (University of Illinois) and subsequent breeding was carried out at Loyola University, Chicago and University of Lausanne. All mice were housed 4–5/cage under a 12:12 h light/dark cycle (lights on from 7am to 7pm). Mice received either NC (Teklad LM-485) or WD (TD88137, Teklad Diets; 42%kcal from fat, 34% sucrose by weight, and 0.2% cholesterol total) (Envigo, Indiana, USA) or WD+10%Inulin (isocaloric with WD) for up to 22 weeks starting at 7 weeks of age. BW were recorded weekly from weaning. All studies mentioned were done using male mice unless stated to avoid confounding effect of hormones with experimenter blinded to both treatment and genotype.

### Human subjects

Informed consent was obtained from all human subjects (biopsies from patients undergoing diagnosis for neuropathy) prior to sural nerve sample collection in strict accordance with the rules and guidelines stipulated according to the protocol, the Swiss legal requirements, the current version of the World Medical Association Declaration of Helsinki and the principles and procedures for integrity in scientific research involving human beings. All experimental protocols were approved by the ethical committee of canton of Vaud (protocol # CER-VD BASEC ID: 2023–01412).

## METHOD DETAILS

### *In vivo* PLX5622 treatment

Microglia/macrophages depletion was performed using PLX5622 (Cat. #1069 HY-113153/CS-0077157, MedChemExpress)^30^. PLX5622 was dissolved in 10% DMSO, 20% RH40 (KolliphorR1070 RH40, Sigma), 5% Tween-20, 65% saline in that order to create a working solution at 6.5mg/ml. Daily intraperitoneal injections were administrated to mice at 65mg/kg body weight for 9 days^30^.

### von Frey Mechanical Sensitivity

Mice were investigated for mechanical allodynia using von Frey filaments^31,32^. Briefly, mice were acclimated to the testing chambers for 60 minutes and were subjected to stimulations with 6 calibrated von Frey filaments (0.16; 0.4; 1; 2; 4; 6; 8 g) (North Coast Medical, California, USA). Filaments were applied for 1 sec with 5 min break between each set of stimulations, with 6 stimulations per filament. Response frequency for each filament was recorded, and 50% threshold was calculated using the Hill equation. A single experienced investigator took all baseline and experimental measurements for these series of experiments while remaining blinded to the genotype and treatment groups. Mice were evaluated in a quiet room, at a constant temperature and acclimated to the von Frey chambers for at least 20 min but not restrained in the chamber any longer than necessary to minimize stress and discomfort-induced behavioral variations. Allodynia was characterized in all behavioral tests as an intense paw withdrawal or licking of the stimulated hind paw^33^.

### Thermal nociception

Mice were investigated using a Plantar Test Apparatus (Hargreaves Method) (IITC Life Science, California USA)^31–33^. Briefly, after acclimation to testing chambers, tests were performed on the plantar surface of mice by a focused, radiant heat light source with a built-in timer displaying reaction time in seconds. A Humane cutoff time of 20 seconds was set at the end of which the heat source shuts off automatically if the animal has not responded, avoiding tissue damage.

### Glucose tolerance test

Overnight (12hrs) fasted mice were given i.p dose of glucose (1g/kg BW) after measuring fasting glucose levels. Blood glucose levels were then monitored using Contour next glucometer for rodents (VitaServ, Switzerland).

### Serum triglycerides, cholesterol, insulin, and leptin measurement

Serum from mice were processed for levels of triglycerides (TR22421, Fisher Scientific), cholesterol (TR13421, Fisher Scientific), insulin, and leptin (EMD Millipore, Massachusetts, USA) using manufacturer’s instructions.

### Dorsal Root Ganglia organotypic culture

DRG organotypic cultures were prepared as described^32^. Briefly, mice were anesthetized with isoflurane before decapitation, and the DRG quickly removed and cultured on an air-interface membrane (Millipore). Cultures were maintained for a week in standard culture medium^34^ replacing every other day in a 37°C and 5% CO_2_ incubator.

### Primary DRG neuronal culture

DRG primary cultures were done as reported before^32^. Briefly, DRG from P10–12 mice were collected in ice-cold advanced DMEM without any supplementation and axotomized. Axotomized DRG were then transferred to a collagenase A/trypsin mix (1.25mg/ml each) and incubated for 30min. Partially digested DRG were then passed through fire polished glass pipettes followed by 3min spin at 3000 g. After careful removal of supernatant, cells were resuspended in DMEM/F-12 with GlutaMAX and 10% FBS and plated onto a poly-l-lysine coated plates. Neuronal cultures were maintained in a 37°C and 5% CO_2_ incubator for 5 days changing above media supplemented with Ara-C (20μM) to inhibit replicative cells every other day. The cells were treated with 10% serum from either NC, WD, or WD-FP mice for 48hrs before electrophysiological studies.

### Acute spinal cord slices preparation

Spinal cord slices were prepared following a previously published protocol^35^. Briefly, animals were deeply anesthetized under isoflurane gas narcosis and manual transcardiac perfusion was performed via injection of 20 mL of cold (4°C) and oxygenated (95% O_2_/5% CO_2_) sucrose-based ACSF containing (in mM): Sucrose 240, KCl 2.5, NaH_2_PO_4_ 1.25, MgCl_2_ 3.5, CaCl_2_ 0.5, NaHCO_3_ 25, ascorbic acid 0.4, Sodium pyruvate 2. Animals were then decapitated, the spinal column opened, and the spinal cord removed into the same sucrose-based ACSF. After careful removal of the spinal roots, the lumbar spinal cord was excised and placed along a medial gutter carved in an 3% agarose block. To prepare coronal slices of spinal cord, the agar block was glued on a vibratome sample plate with the spinal cord oriented vertically (Leica VT1200S) and the vibratome slicing chamber was filled with cold and oxygenated sucrose-based ACSF. 250μm slices obtained were immediately transferred in ACSF solution maintained at 32°C, equilibrated with 95% O_2_/5% CO_2_, and containing (in mM): NaCl 126, KCl 1.6, NaH_2_PO_4_ 1.1, MgCl_2_ 1.4, CaCl_2_ 2.4, NaHCO_3_ 26.2, Glucose 11 (300 ± 5 mOsm). For electrophysiology, after a minimal 30min resting period, slices were transferred into the recording chamber under an upright epifluorescence microscope (BX51WI, Olympus, Japan) mounted on a motorized stage coupled to a micromanipulator (MPC-325, Sutter Instrument, USA) and equipped with an Evolve EMCCD camera (Teledyne Photometrics Technology, USA) to visualized neurons of the substancia gelatinosa.

### Patch-clamp recordings

Neuronal activity, properties and excitability of spinal cord neurons from the substancia gelatinosa area and DRG primary neurons were assessed via patch-clamp recording under whole-cell configuration using a MultiClamp 700B amplifier coupled to a 1440A Digidata digitizer (Molecular Devices). Patch pipets were made from borosilicate glass capillaries (GC150F-7.5; Warner Instruments) using a pipet puller (DMZ Universal Electrode Puller; Zeitz) and filled with intracellular solution containing (in mM): K-Gluconate 130, NaCl 5, MgCl2 1, Na-Phosphocreatinine 10, HEPES 10, EGTA 0.2, MgATP 4, Na2GTP 0.5 (280 ± 5 mOsm, pH 7.3). Extracellular recording ACSF solution contained (in mM): NaCl 126, KCl 1.6, NaH2PO4 1.1, MgCl2 1.4, CaCl2 2.4, NaHCO3 26.2, Glucose 11 (300 ± 5 mOsm, equilibrated with 95% O2/5% CO2 and maintained at 32–34°C). Neurons exhibiting an access resistance over 25 MΩ or changed by more than 20% during the recording were discarded from analysis. A hyperpolarization step (−5 pA, 500 ms) was applied every 30 s to assess membrane resistance. Resting membrane potential and resistance were monitored over time in current-clamp mode (i=0) and calculated over 5min after a 10min baseline period following membrane break in. Neuronal excitability was determined by injecting series of 1s depolarizing current pulses (25 to 150 pA with 25 pA increment for spinal cord neurons or 100 to 1000 pA with 100 pA increment for DRG primary neurons) from resting membrane potential. For IV curves, cells were held at −50 mV and stepped from −100 mV to +80 mV in 10 mV intervals. Signals were filtered at 2 kHz, digitized at 10 kHz, and collected online with a pClamp 10 data acquisition system (Molecular Devices).

### Ex-vivo calcium imaging of cultured DRG using two-photon microscopy

Cultured DRG ganglia (see DRG organotypic culture section) were transfered in the climate chamber surrounding the microscope, keeping the whole stage of the microscope and the objectives at 34°C. Ganglia were placed in a submerged-type recording chamber under the microscope (JG-23W/HP, Warner Instruments, Hamden, USA), held down with a nylon mesh harp, and continuously superfused at a flow rate of 2 mL/min with oxygenated ACSF solution containing (in millimolars): 126 NaCl, 1.6 KCl, 1.1 NaH2PO4, 1.4 MgCl2, 2.4 CaCl2, 26 NaHCO3, and 11 glucose (295 to 305 mOsm). A peristaltic pump (Ismatec Ism831C) allowed the perfusion of the microscope chamber with ACSF and pharmacological compounds. The imaging system was composed of an upright Leica TCS SP8 DIVE multi-photon microscope (Mannheim, Germany) with an InSight X3 and a Mai Tai both tunable lasers (Spectra Physics) and three non-descanned hybrid 4Tune detectors plus a photomultiplier detector. The resonant scanner imaging system was used at a frequency of 8000Hz. Both tdTomato and GCaMP6f imaging was performed using a wavelength of 920 nm for excitation and detected between 570–670 nm and 490–525 nm respectively. All images were acquired with a 16× multi-immersion objective (HC FLUOTAR L N.A. 0.6 FWD 2.5 mm). GCaMP6f and tdTomato signals were acquired in parallel with no time delay. To correct for potential photobleaching/motion artefacts and/or inter-ganglia promoter expression variation, we divided GCaMP signal by tdTomato signal (G/R ratio) offline. For comparison of individual neuronal response to tested pharmacology, we normalized this GCaMP/tdTomato signal ratio to basal fluorescence (F0). All neuronal responses from the same diet condition were then pooled together.

### Immunohistochemistry

Tissues were fixed overnight in buffered formalin before washing with PBS and stored in 30% sucrose solution untill sectioning using a cryostat. Sections were washed three times with PBS. Nuclei were counterstained with DAPI, and coverslips were mounted using an antifade reagent. Imaging was performed using a THUNDER Imaging System (Leica Microsystems), and image analysis was conducted using ImageJ software.

### 16S sequencing

DNA from caecal samples was collected and DNA extracted using a slightly modified protocol for the Maxwell RSC PureFood GMO and Authentication kit (#AS1600) and the Maxwell RSC 48 extraction robot (#AS8500). In brief 100 mg of caecal content were weighed into Qiagen PowerBead Pro tubes (#19301) mixed with 1ml of CTAB buffer, incubated for 5 min at 95°C prior to vortexing for 10 min at full speed on the Votex Genie 2 with a 24-tube adaptor (#13000-V1–24) prior to vortexing for 10 min at 14’000g. The supernatant was then incubated for 10 min at 70°C with 40μl of Proteinase K and 20μl of RNAse A provided in the Maxwell kit before continuing with the standard protocol of the Maxwell kit. The extracted DNA was sent to Novogene, where DNA was amplified using PCR targeting the V4-V5 regions of the 16S rRNA gene using primers 515F and 907R on an Ilumina NovaSeq 6000 platform.

Taxonomy was inferred using the Dada2 pipeline using the Silva Database version 138.1 adapted for Dada2 using the Standard Dada2 pipeline. Biostatistical analyses were performed using the phyloseq pipeline. Alpha diversity was based on observed ASV count, the Chao1, Shannon and Inversed Simpsons indeces. Beta diversity was calculated using the Bray-Curtis Dissimilarity Index. Statistical comparisons were performed using the Wilcoxon rank sum test for alpha diversity and Permanova for beta diversity, respectively. Differences in relative abundances in between groups were estimated using DeSeq2 with a significance level fixed at 0.01 and the Wald test.

### SCFA Quantification

SCFA levels from serum and cecal contents were analysed as previously published^31,36,37^. All samples were analyzed in triplicate by the Metabolomic platform of University of Lausanne and an internal control was used to evaluate the interassay variability.

### Body composition

Measurements of lean, fluid, and fat mass were performed using Minispec LF50 nuclear magnetic resonance (NMR) analyzer (Bruker Corporation, Billerica, MA).

### Enrichment of transcripts from myeloid cell lineage in adipose tissue

Adipose from RiboTag+/+:LysMcre+/− mice were freshly harvested for isolation of RNA in translation. To isolate RNA associated with HA-tagged ribosomes in myeloid cells, immunoprecipitation (IP) followed by mRNA purification following the procedure published by Sanz et al. was used^32,38^. Briefly, adipose tissue was homogenized in homogenization buffer and supernatant removed after centrifuging at 10,000g for 10 min at 4°C. Supernatant was incubated at 4°C with anti-HA antibody (Biolegend, #901513) at 1:150 dilution for 4hrs on a gentle spinner. This is followed by an overnight incubation at 4°C on a gentle spinner with above sample transferred to tube containing magnetic beads (Pierce A/G magnetic beads, California, USA). Supernatant form the samples were collected and beads were washed with high salt buffer, 3 times,10 min each at 4 ° C on spinner. After final wash, lysis buffer (RNeasy Micro Kit, Qiagen, Maryland, USA) with β-mercaptoethanol (10μl/ml) was added to elute the mRNA. Total RNA from the IP’ed polysomes was eluted using RNeasy Micro Kit (Qiagen, California, USA) following manufacturer’s instructions and quantified with Quant-iT RiboGreen RNA Assay kit (Invitrogen, California, USA) and Agilent Bioanalyzer. Eluted RNA was then processed for RNASeq.

### Flow cytometry

Cells dissociated from the DRG, spinal cord, and whole SN were stained with fluorescently conjugated antibodies (CD45-BB515, CD11b-RB705, MHCII-APC-Cy7, F4/80-Alexa647, and CD206-PE) (BioLegend, California, USA) as previously described^31,39^. Briefly, dissociated single cells from tissues were incubated in blocking solution (5% horse serum in PBS) for 30 min on ice. After pelleting the cells at 300 g for 5 min, above-mentioned fluorescently labelled antibodies were assayed at 1:500 dilution and incubated for 1 h on ice. Cells were then washed and resuspended in blocking buffer before analyzing. Cells that were positive for CD45 were sorted from other dissociated cells for further analysis. Flow cytometry data were acquired using a BD FACS Aria III (BD Biosciences, California, USA) and data analyzed using FlowJo (BD Biosciences, California, USA).

### Cytokine assay

Serum cytokine levels and pro-/anti-inflammatory state of mice were assessed using the LEGENDplex^™^ Mouse Macrophage/Microglia Panel (13-plex) (Cat# 740846, BioLegend), according to the manufacturer’s protocol. The samples were analyzed using a cytoFLEX (Beckman Coulter, USA). Data acquisition was performed with LEGENDplex^™^ Data Analysis Software.

### RNA isolation, cDNA library construction and illumina sequencing

RNASeq was performed as done before^31^. Briefly, total RNA was extracted from DRG of mice with Arcturus PicoPure RNA isolation kit (Applied Biosystems). Four biological replicates were used for each group. Total RNA was quantified by Qubit and assessed for quality on an Agilent Bioanalyzer using Total RNA Pico Chip. Total RNA samples that passed QC were used as input for library construction. Full-length cDNA synthesis and amplification were carried out with the Clontech SMART-Seq v4 Ultra Low Input RNA Kit. Subsequently, Illumina sequencing libraries were prepared from the amplified full-length cDNA with the Nextera XT DNA Library Preparation Kit. Prior to sequencing, the prepared libraries were quantified with Qubit and validated on a Bioanalyzer with a High Sensitivity DNA chip. The sequencing of the libraries was conducted on an Illumina NextSeq 500 NGS System. Single 75 bp reads were generated with dual indexing. RNA-seq Analysis done with STAR and DESeq2. The quality of reads, in FASTQ format, was evaluated using FastQC. Reads were trimmed to remove Illumina adapters from the 3′ ends using cutadapt51. Trimmed reads were aligned to the Mus musculus genome (mm10) using STAR52. Read counts for each gene were calculated using htseq-count in conjunction with a gene annotation file for mm10 obtained from Ensembl (http://useast.ensembl.org/index.html). Normalization and differential expression were calculated using DESeq2 that employs the Wald test. The cutoff for determining significance was a q-value less than 0.05 using the Benjamini-Hochberg method and a log-fold change less than −0.6 or greater than 0.6. Pathway analyses were performed using Metascape (http://metascape.org) to identify enriched pathways among genes with significant differential expression. Genes with significant changes in expression were divided into upregulated and downregulated genes and analyzed separately. Pathways with q-values less than 0.05 were considered significant.

### FFAR2 expression in human and mouse

For the analysis of human and mouse data, single-cell RNA-seq datasets were downloaded from the cellxgene databases (https://tabula-sapiens.sf.czbiohub.org; https://tabula-muris.sf.czbiohub.org) and analyzed.

### Quantitative PCR

mRNA was extracted from sorted CD45^+^ cells using Arcturus PicoPure RNA Isolation Kit (ThermoFisher, Massachusetts, USA) before generating cDNA using High-Capacity cDNA Reverse Transcription Kit (ThermoFisher, Massachusetts, USA). For all genes of interest, qPCR was performed using Sybr green-based assay (Roche, Indiana, USA) using IDT primers (IDT technologies, Iowa, USA). 18s was used to normalize data and quantification was done using ΔΔCT method with control group’s mean value set at 100%.

## QUANTIFICATION AND STATISTICAL ANALYSIS

All data are represented as mean±S.E.M. Analysis were performed using Graphpad prism 10.4.1. For single group comparisons 2-tailed t-test was used as appropriate with multiple comparisons performed using ANOVA. For repeated measures, 2-way ANOVA was used and p value less than 0.05 considered significant.

## Supplementary Material

Supplementary Files

This is a list of supplementary files associated with this preprint. Click to download.

- supp1.jpg
- supp2.jpg

## Data Availability

Further information and requests for resources and reagents should be directed to the Lead contact, Virginie Mansuy-Aubert (virginie.mansuy-aubert@unil.ch)

## Highlights

- Inulin attenuates WD-induced sensory hypersensitivity and neuronal hyperexcitability
- Inulin restores WD-induced metabolic and microbial alterations
- Serum factors, but not SCFAs, modulate dorsal root ganglia neuronal excitability in a diet-dependent manner
- Inulin remodels adipose tissue immunity and systemic cytokines to modulate sensory function
- Adipose myeloid FFAR2 mediates gut–immune crosstalk underlying sensory dysfunction
- Inulin modulates neuroimmune interactions in sensory tissues to improve neuronal function

## Significance

Somatosensory system dysfunction is an underrecognized complication of obesity and Western-style diets, leading to chronic pain and sensory loss. Current therapies are limited because the mechanisms linking diet, metabolism, and nerve health remain complex and unclear. Here we show that supplementing a Western diet with the fermentable complex carbohydrate, inulin, both prevents and reverses sensory deficits in mice by restoring microbial short-chain fatty acid production and reprogramming immune cells in adipose tissue, thereby reducing systemic and neural inflammation. These findings uncover a previously unrecognized gut– adipose–nerve communication axis that governs sensory function. Given declining fiber intake worldwide and rising rates of obesity, our results highlight dietary fiber as an accessible strategy to preserve nerve health in obesity-associated neuropathy.
